# The relationship between frailty in older adults and anxiety and depression in china: propensity score matching and network analysis

**DOI:** 10.3389/fpsyt.2025.1596015

**Published:** 2025-07-30

**Authors:** Yinglin Li, Ling Zhao, Doudou Lin, Xinmei Wang, Chunlong Zhang, Jiali Zhou, Zhongxiang Cai

**Affiliations:** ^1^ Department of Nursing, Renmin Hospital of Wuhan University, Wuhan, China; ^2^ Department of Geriatrics, Renmin Hospital of Wuhan University, Wuhan, China

**Keywords:** frailty, older adults, anxiety, depression, network analysis

## Abstract

**Objective:**

Anxiety and depression are common mental disorders in the elderly. Concurrent frailty may lead to worse clinical outcomes. This study examined the network structures of anxiety and depression in frail and non-frail older adults.

**Methods:**

The Center for Epidemiologic Studies Depression Scale-10 (CESD-10) and the Generalized Anxiety Disorder Scale-7 (GAD-7) were used to measure depressive and anxiety symptoms, respectively. Following propensity score matching (PSM), 877 frail elderly individuals were matched with 877 non-frail elderly individuals. Central (influential) and bridge symptoms were estimated using the expected influence (EI) and bridge expected influence (bridge EI), respectively. Network stability was assessed using the case-dropping bootstrap method.

**Results:**

Based on the NCT results, there were no significant differences in the comparison of the network models between the non-frailty group and the frailty group in terms of global strength (7.175 vs. 7.136, S = 0.039, P = 0.802) and network structure (M = 0.137, P = 0.703). There were also no significant differences in edge weights between the networks of the two groups (P > 0.05).

**Conclusion:**

NCT results showed no significant difference in the network structure of anxiety and depression between frail elderly and control groups. A slight decrease in network strength was observed in non-frail elderly but was not statistically significant. Both groups showed similar characteristics in bridging symptoms, central symptoms, overall strength, and network structure. Interventions for anxiety and depression are equally beneficial for both frail and non-frail elderly.

## 1 Introduction

Population aging represents a significant challenge confronting numerous countries and regions globally, which exerts an extensive and profound impact on human society. The Bulletin on the Development of the National Aging Undertaking indicates that in China, the population aged 60 and above has surpassed 280 million (19.8% of the total population) by the end of 2022 (1). This reflects that the degree of population aging in China is continuously intensifying, and the long-term balanced development of the population is facing pressure. How to improve the life quality of older adults, solve the predicament of population aging, and promote the development of health care for older adults has become a hot topic that is widely concerned by all sectors of society.

The health conditions of older adults change with increasing age. Problems such as the decline of cognitive, motor and sensory functions, unbalanced dietary nutrition, decreased physical immunity and increased underlying diseases have become increasingly prominent. Coupled with significant changes in social roles, older adults tend to have a series of psychological problems such as loneliness, depression and hypochondria. Anxiety and depression are the two most frequent psychological disorders in older adults. Research indicates that in China, the overall prevalence rate of depressive symptoms among older adults is 20% (2). Elderly individuals living in an empty nest are at a heightened risk of depression, with a prevalence rate of as high as 38.6% (3). The potential threat that anxiety poses to the mental and physical health of the elderly warrants significant attention. A 2022 study revealed that 65% of older adults reported experiencing symptoms of anxiety, and nearly a quarter showed anxiety levels comparable to patients diagnosed with generalized anxiety disorder (4). A study shows that the prevalence rate of anxiety symptoms among the elderly in primary medical care in China is 21.1% (5). Among the elderly population, 8.7% exhibited comorbid symptoms of depression and anxiety (6).

“Frailty” was initially proposed by O’Brien et al. in the 1960s as a term to depict the improper responses of elderly people to a series of adverse internal and external events (7). Until the 21st century, Fried et al. formulated a more precise definition of frailty. Currently, it is believed that frailty pertains to the decline or abnormality of the physiological functions of the elderly. This leads to the inability to sustain or lose normal physiological balance when the body responds to external traumas and stresses, thereby giving rise to the occurrence of clinical events even with minor stimuli (8). In a report covering 62 countries worldwide, the prevalence of frailty among community-dwelling persons ranged from 11% among those who were 50 to 59 years of age to 51% among those who were 90 years of age or older (9). One meta-analysis of 14 studies with a total of 81,258 participants reported a pooled frailty prevalence of 10% (95% CI 8–12) among Chinese adults in the community aged 65 years and older (10). Other studies have also shown that the prevalence of frailty was 8·9% in participants aged 65 years and older (11).

Due to multiple factors, such as the escalating prevalence of the elderly, the deterioration of neural cell functions, physical disability, and living alone, older adults are prone to mental illnesses. Anxiety and depression are common mental and psychological disorders among the elderly. If they occur with frailty concurrently, it may give rise to more adverse clinical outcomes, such as death (12).

Network analysis represents a new statistical method that has been extensively utilized recently to construct orderly spatial networks and simultaneously clarify the relationship between multiple symptoms (13–15). Within the framework of network models, a single symptom is assigned as a node, and the position of each node indicates the significance of each corresponding symptom (16). The interaction between symptoms is defined as the edge, and the thickness of the edge denotes the magnitude of the symptom relationship (17). Furthermore, the node centrality index manifests the connectivity of a variable to all the remaining variables within the network. For example, the most prominent symptoms of a network model are identified using the expected influence (EI), and the symptom influence and the interconnection of symptom clusters are illuminated using the bridge EI (17). Network analysis is inclined to contribute to the ascertainment of critical factors that function as bridges between psychological factors associated with the elderly and frailty (18). These bridges within the network can serve as intervention points to facilitate the propagation of intervention effects or obviate the risk of negative psychological contagion, thus evading adverse health consequences (19).

As of now, no published research has emerged regarding the network analysis of anxiety and depression in older adults with frailty. To bridge this gap and enhance the health outcomes of frail older adults, a comparison of the network structure of anxiety and depression between older adults with frailty and those without frailty (the control group) was conducted. It is postulated that the network structure of depressive symptoms in frail elderly patients would significantly differ from that of the control group.

As of now, no published research has emerged regarding the network analysis of anxiety and depression in older adults with frailty. Given that anxiety and depression are bidirectional risk factors that mutually influence each other (20), this study aims to investigate differences in the symptom networks of anxiety and depression between frail and non-frail elderly populations. Based on data from the China Longitudinal Healthy Longevity Survey (CLHLS), propensity score matching and network analysis were employed to achieve this objective. The primary hypothesis posits that significant differences exist in the structure of anxiety and depression symptom networks between frail and non-frail older adults. Secondary hypotheses focus on intergroup variations in symptom association strength, network stability, subnetwork configurations, and predictive effects. By elucidating how frailty influences the interaction dynamics of anxiety and depression symptoms, this research seeks to inform targeted interventions designed to enhance mental health outcomes among both frail and non-frail elderly individuals.

## 2 Methods

### 2.1 Participants and procedures

Data were obtained from the China Longitudinal Healthy Longevity Survey (CLHLS 2017-2018). CLHLS is a nationwide prospective cohort study focusing on older adults who live in Chinese communities. CLHLS initiated a baseline survey in 1998 and performed seven follow-up surveys in 23 provinces of China, covering about 85.0% of the national population. These surveys took place in 2000, 2002, 2005, 2008-2009, 2011-2012, 2014, and 2017-2018. Trained interviewers administered the surveys in participants’ homes (21). To ensure the representativeness of the sample, CLHLS employs a targeted, disproportionate, and multi-stage random sampling method, with a particular emphasis on individuals aged 65 and above. The CLHLS study was approved by the Institutional Review Board of Duke University (Pro00062871) and the Biomedical Ethics Committee of Peking University (IRB00001052-13074) (21). Further details regarding CLHLS have been reported elsewhere, and the overall data quality is generally considered satisfactory. The CLHLS 2017–2018 dataset is free, public, and open.

The inclusion criteria are as follows: (1) age ≥ 65 years; (2) complete items for basic demographic information, measurement of frailty, depression, and anxiety.Therefore, participants with missing values in any of these key variables—including frailty status, CESD-10, GAD-7, or covariates used in the propensity score model—were excluded from the analysis. A complete-case analysis approach was adopted. **Supplementary Figure S1** shows the sample selection procedure.

### 2.2 Measures

Sociodemographic data were collected, including age, gender, years of education, marital status (specifically whether widowed), household registration status, insurance status, living arrangements, sleep duration, life satisfaction (“How would you rate your current life?”), smoking history, alcohol consumption status, dietary preferences, past exercise habits, and current exercise habits.

Frailty is assessed using a modified version of the SOF index, which evaluates three criteria: (1) underweight (BMI < 18.5 kg/m²); (2) muscle strength (inability to stand up from a chair without arm support); and (3) reduced energy (a positive response to the question “Have you been limited by health problems in the past six months?”) (22, 23). Based on the total score, individuals can be classified into three categories: robust (0 points), prefrail (1 point), and frail (2 or 3 points). In this study, participants classified as robust and prefrail were considered to be in non-frail states, in accordance with previous studies that adopted similar classification criteria in community-dwelling older adults (24).

Depressive symptoms were evaluated using the Center for Epidemiologic Studies Depression Scale-10 (CESD-10), which has been verified among older adults in China (25, 26). The CESD-10 is a 10-item Likert scale with responses ranging from 0 (never) to 3 (always), yielding a total score between 0 and 30. More severe depressive symptoms are indicated by higher scores (27). According to previous studies (28), a score of ≥10 suggests the presence of depressive symptoms, while a score of ≥20 suggests severe depressive symptoms. In this study, the Cronbach’s alpha coefficient was 0.78, representing an acceptable level of internal consistency.

Anxiety symptoms were evaluated using the Generalized Anxiety Disorder Scale-7 (GAD-7), which assesses the frequency of anxiety symptoms in the past two weeks. The GAD-7 is a 4-point Likert scale of 7 items and individual item scores range from 0 (not at all) to 3 (almost every day), leading to a total possible score of 0-21. More severe anxiety symptoms are indicated by higher total scores (29). The cut-off scores of 5, 10, and 15 correspond to mild, moderate, and severe levels of anxiety, respectively (29, 30). In this study, the Cronbach’s α coefficient was 0.93, indicating excellent internal consistency.

### 2.3 Statistical analysis

#### 2.3.1 Propensity score matching and univariate analysis

To minimize demographic differences between frail and non-frail elderly individuals, propensity score matching (PSM) in R (version 5.4.1) was conducted using the MatchIt package (version 4.5.5) (31). The nearest neighbor matching with a caliper of 0.1 and a ratio of 1:1 was employed (32). PSM is a statistical technique used to reduce selection bias and achieve a covariate balance between groups in observational studies (32, 33). A logistic regression model was used to estimate the propensity scores. Covariates were selected based on their significant differences as identified by chi-square tests and independent sample t-tests. Specifically, covariates comprised age, gender, education level, marital status (with a focus on whether participants were widowed), living arrangements, daily sleep duration, medical insurance type, life satisfaction, smoking status, alcohol consumption, taste preference, as well as past and current exercise habits. In addition to statistical differences, the selection of covariates was also supported by prior empirical studies that identified these sociodemographic and lifestyle factors as significant correlates of frailty among older adults in China (34, 35).

#### 2.3.2 Network analysis

Network analysis was performed using R (version 4.4.1). The R packages bootnet (version 1.6.0) and qgraph (version 1.9.8) (17) were employed for network estimation and visualization. Both CESD-10 and GAD-7 are Likert scales, and Spearman’s correlation was used to estimate the edges between items (36). The enhanced least absolute shrinkage and selection operator (ELASSO) was applied to assess the significance of these edges and minimize spurious connections (37). The Extended Bayesian Information Criterion (EBIC) was used to guide model selection, with a tuning parameter *γ* = 0.5 to control for sparsity (38). In this network, each item is represented as a node, and each pairwise association between items is represented as an edge. A stronger correlation is indicated by a thicker edge, while positive and negative correlations are indicated by purple and red edges, respectively.

The bootnet package (version 1.6.0) (17) was utilized to evaluate the stability and accuracy of network models. Accuracy is the extent to which sample estimates reflect the true population parameters, evaluated by plotting the 95% confidence intervals (CIs) of edge weights based on 1500 bootstrap samples. Narrower CIs indicate higher accuracy. Stability is quantified and visualized using the correlation stability coefficient (CS-C), computed with 4000 bootstrap samples. A CS value of 0.70 indicates the maximum acceptable level of sample reduction, while a CS value of >0.50 is considered acceptable, with a minimum threshold of 0.25. A bootstrap difference test was employed to evaluate the stability of node EI and edge weights, where a larger black area signifies more significant differences.

To compare differences in the network structure of depressive and anxiety symptoms between the two groups, a network comparison test (NCT) was conducted using the “NetworkComparisonTest” package (version 2.2.2) (39).

## 3 Results

### 3.1 Study sample

A total of 8,369 non-frail and 886 frail elderly individuals were initially screened. Following propensity score matching (PSM), 877 frail elderly individuals were matched with 877 non-frail elderly individuals. **Table 1** gives a summary of the clinical and demographic characteristics of the matched study sample. The standardized mean difference (SMD) of the matched demographic variables was 0.004, indicating excellent balance between the groups. **Supplementary Figure S2** illustrates the distribution and histogram of the propensity scores.

**Table 1:** Characteristics of participants included in the study (N = 1754).

Characteristics	Non-frailty (N = 877)	Frailty (N =877)	*t/χ^2^*	*P*
	Mean ± SD or n (%)	Mean ± SD or n (%)		
Age (years)	92.75 ± 9.55	92.92 ± 9.57	-0.382	0.702
Education level (years)	2.08 ± 3.57	2.27 ± 3.99	-1.073	0.284
Daily sleep duration (hours)	7.46 ± 2.44	7.64 ± 2.71	-1.444	0.149
Gender			0.044	0.834
Male	257 (29.3%)	262 (29.9%)		
Female	620 (70.7%)	615 (70.1%)		
Living arrangement			0.257	0.879
Family	712 (81.2%)	704 (80.3%)		
Living alone	95 (10.8%)	101 (11.5%)		
Nursing home	70 (8.0%)	72 (8.2%)		
Birthplace			1.424	0.233
Urban	255 (29.1%)	279 (31.8%)		
Rural	622 (70.9%)	598 (68.2%)		
Widowed			0.003	0.955
Yes	198 (22.6%)	200 (22.8%)		
No	679 (77.4%)	677 (77.2%)		
Smoking			0.108	0.742
Yes	79 (9.0%)	84 (9.6%)		
No	798 (91.0%)	793 (90.4%)		
Drinking			0.927	0.336
Yes	65 (7.8%)	77 (9.0%)		
No	812 (92.2%)	800 (91.0%)		
Taste preference			3.037	0.694
Mild taste	640 (73.0%)	634 (72.3%)		
Salty foods	146 (16.6%)	141 (16.1%)		
Sweet foods	41 (4.7%)	53 (6.0%)		
Spicy foods	16 (1.8%)	11 (1.3%)		
Raw and Cold Foods	1 (0.1%)	2 (0.2%)		
None of the Above Taste Preferences	33 (3.8%)	36 (4.1%)		
Currently exercise regularly			0.000	1.000
Yes	103 (11.7%)	102 (11.6%)		
No	774 (88.3%)	775 (88.4%)		
Used to exercise regularly			0.000	1.000
Yes	216 (24.6%)	215 (24.5%)		
No	661 (75.4%)	662 (75.5%)		
Medical payers			1.490	0.914
Urban medical insurance	191 (21.8%)	209 (23.8%)		
Rural cooperative medical insurance	252 (28.7%)	256 (29.2%)		
Commercial health insurance	3 (0.3%)	3 (0.3%)		
No medical insurance	431 (49.2%)	409 (46.6%)		
Life satisfaction	2.28 ± 0.77	2.25 ± 0.83	0.776	0.438
CESD-10 total score	10.34 ± 4.01	11.47 ± 4.14	-5.798	**<0.000**
Depression symptoms (CESD-10≥10)	544 (62.0%)	641 (73.1%)	23.974	**<0.000**
GAD-7 total score	1.49 ± 2.87	2.12 ± 3.40	-4.186	**<0.000**
Anxiety symptoms (GAD-7≥5)	108 (12.3%)	173 (19.7%)	17.357	**<0.000**

CESD-10, The Center for Epidemiologic Studies Depression Scale-10; GAD-7, The Generalized Anxiety Disorder Scale-7.

### 3.2 Network structures in widowed versus non-widowed groups

#### 3.2.1 Network structure and centrality symptoms

**Figure 1:**
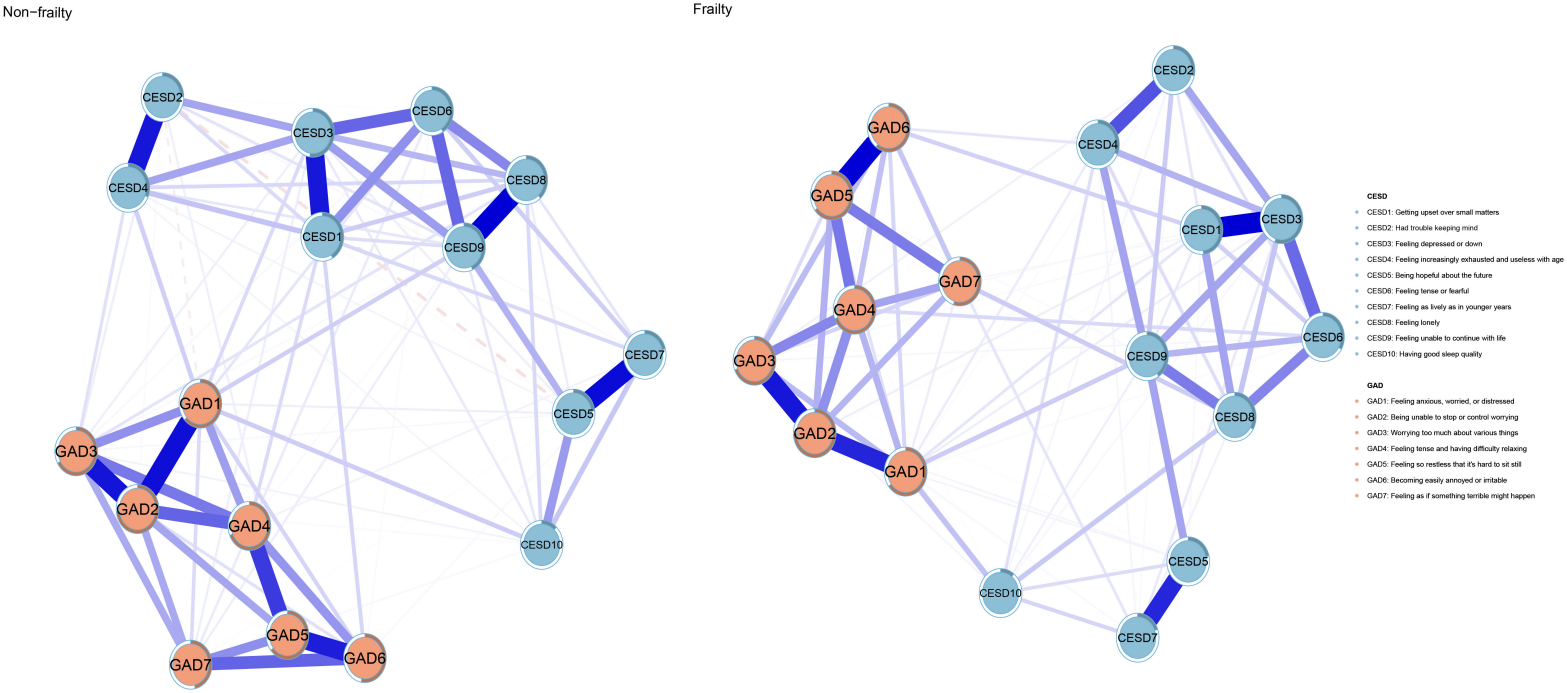
The network structure of anxiety and depression in frail and non-frail elderly people.

**Figure 2:**
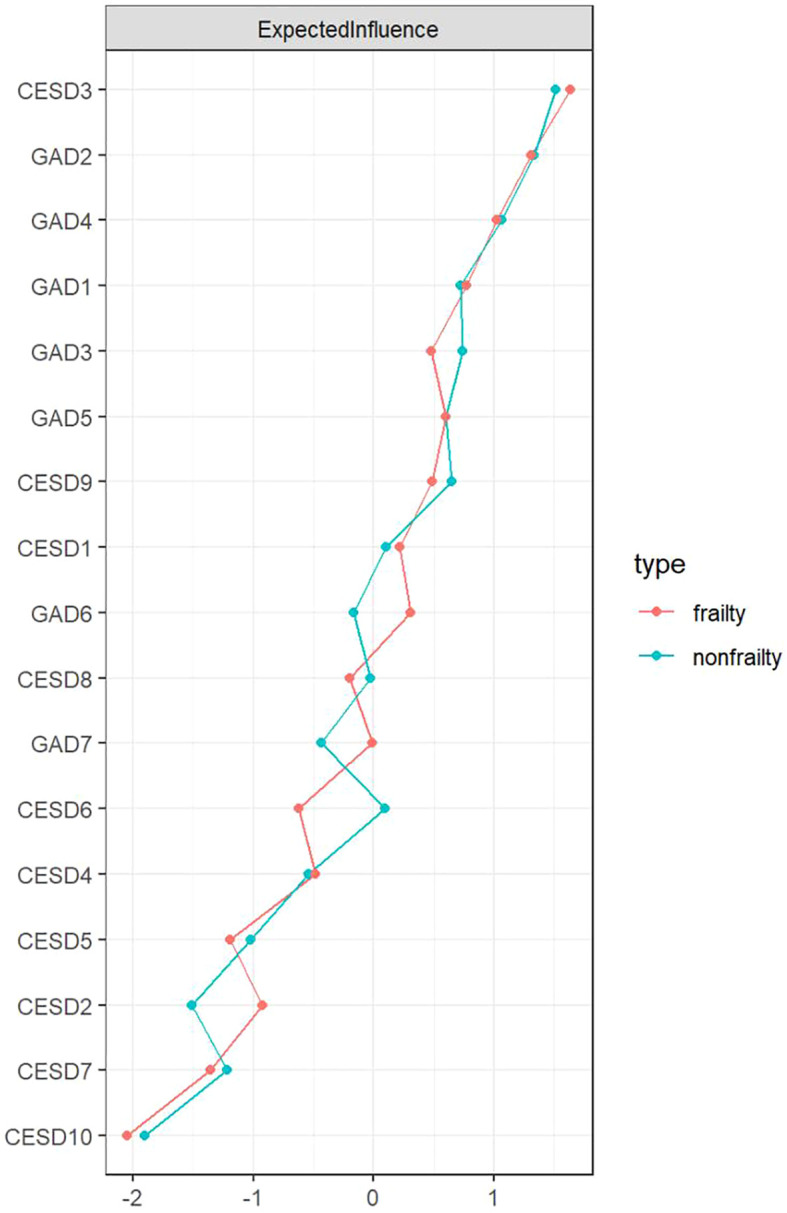
The standardized value (z-score) of EI for each node in the frailty and non-frailty groups.

**Figure 1** depicts the network architectures of depression and anxiety in the frailty and non-frailty groups. In the network of the “non-frailty” group, 89 out of 136 edges (65.4%) have non-zero weights, while in the network of the “frailty” group, 84 out of 136 edges (61.8%) have non-zero weights. Both networks are relatively dense. **Supplementary Table S1** shows the weights of all edges in the two networks. In the network of the “frailty” group, CESD3 (“Feeling depressed or down”; EI: 1.20), GAD2 (“Being unable to stop or control worrying”; EI: 1.13), and GAD4 (“Feeling tense and having difficulty relaxing”; EI: 1.07) are the main symptoms; in the network of the “non-frailty” group, CESD3 (“Feeling depressed or down”; EI: 1.17), GAD2 (“Being unable to stop or control worrying”; EI: 1.13), and GAD4 (“Feeling tense and having difficulty relaxing”; EI: 1.07) are the main symptoms. **Figure 2** gives the comparison of node EI between the non-frailty group and the frailty group. The average predictability of the two groups is 0.459 (frailty group) and 0.450 (non-frailty group), respectively. Among them, GAD2 (frailty group; R2: 0.746) and GAD2 (non-frailty group; R2: 0.731) have the highest predictability. **Supplementary Tables S2** and **S3** display the specific values of node EI and BEI and the specific values of predictability in the two groups.

#### 3.2.2 Bridging symptoms

**Figure 3:**
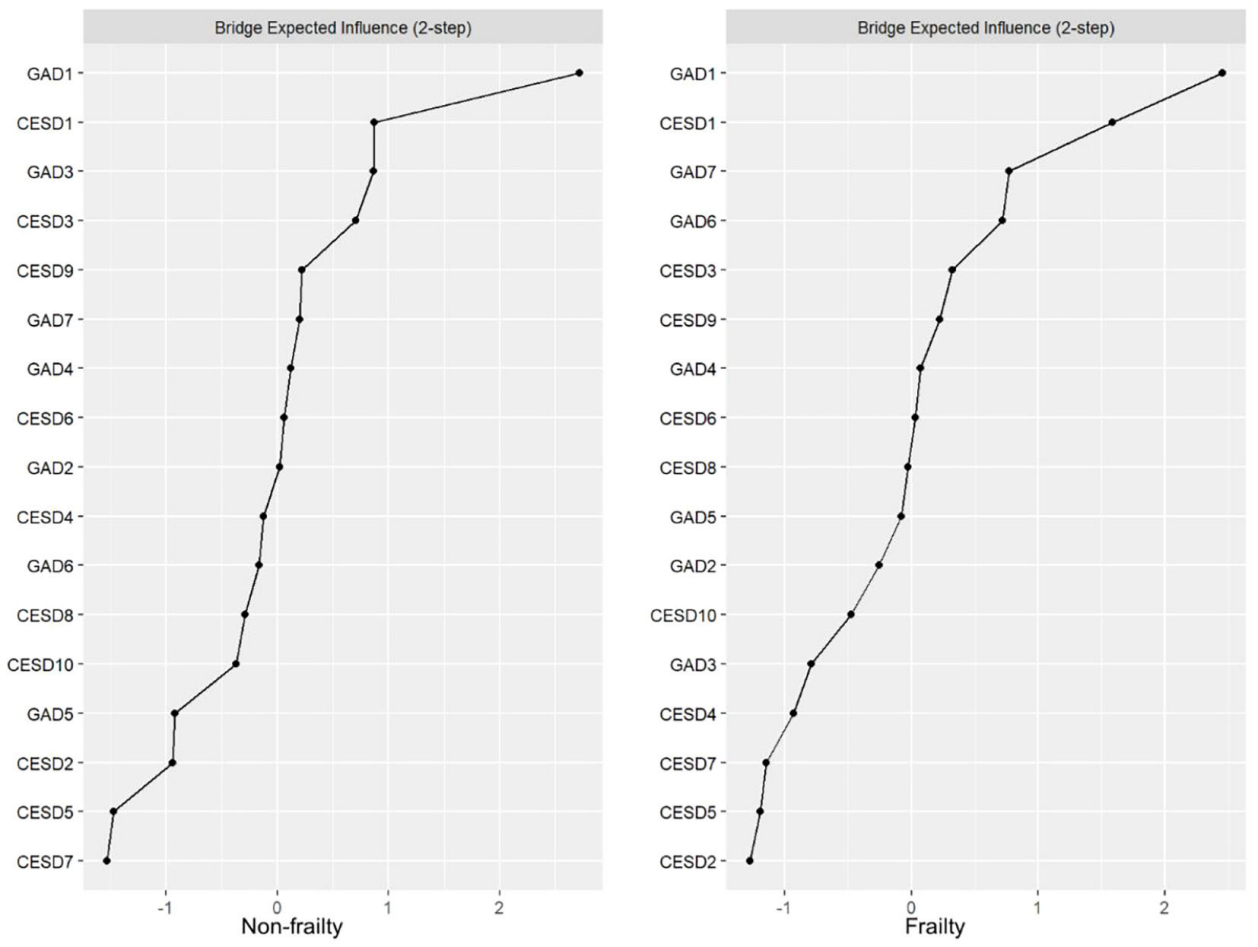
Bridge centrality indices (z-score) of the non-frailty and frailty groups.

**Figure 3** presents the comparison of BEI between the non-frailty group and the frailty group. In the network of the “frailty” group, the crucial bridging symptoms are GAD1 (“Feeling anxious, worried, or distressed”; BEI: 0.52), CESD1 (“Getting upset over small matters”; BEI: 0.42), and GAD7 (“Feeling as if something terrible might happen”; BEI: 0.32). Among them, GAD1 is most tightly linked to CESD10 (“Having good sleep quality”) in the depression community (edge = 0.095); CESD1 is most closely associated with GAD6 (“Becoming easily annoyed or irritable”) in the anxiety community (edge = 0.070); and GAD7 is most strongly connected to CESD8 (“Feeling lonely”) in the depression community (edge = 0.078). In the network of the “non-frailty” group, the key bridging symptoms are GAD1 (“Feeling anxious, worried, or distressed”; BEI: 0.52), CESD1 (“Getting upset over small matters”; BEI: 0.31), and GAD3 (“Worrying too much about various things”; BEI: 0.31). Among them, GAD1 is most closely related to CESD10 (“Having good sleep quality”) in the depression community (edge = 0.068); CESD1 is most strongly associated with GAD6 (“Becoming easily annoyed or irritable”) in the anxiety community (edge = 0.055); and GAD3 is most tightly connected to CESD4 (“Feeling increasingly exhausted and useless with age”) in the depression community (edge = 0.041).

#### 3.2.3 Network stability and accuracy

**Supplementary Figure S3** reveals the narrow bootstrap 95% CI of the estimated edge weights, signifying excellent accuracy. **Supplementary Figure S4** indicates that EI and BEI exhibit considerable stability. Herein, the CS coefficients of EI and BEI in the frailty group are 0.750 and 0.594, respectively; those in the non-frailty group are 0.750 and 0.517, respectively. The comparison of CS coefficients between groups is presented in **Table 2**. The bootstrapped difference test demonstrates that the majority of edge weights and node EIs show statistically significant differences, suggesting that the main results are reliable (**Supplementary Figure S5**).

**Table 2:** Correlation stability (CS) coefficients for expected influence (EI) and bridge expected influence (BEI) in frailty and non-frailty groups.

Group	EI (CS-coefficient)	BEI (CS-coefficient)
Frailty	0.750	0.594
Non-frailty	0.750	0.517

#### 3.2.4 Comparison of the two network models

Based on the NCT results, there were no significant differences in the comparison of the network models between the non-frailty group and the frailty group in terms of global strength (7.175 vs. 7.136, S = 0.039, *P* = 0.802) and network structure (M = 0.137, *P* = 0.703). There were also no significant differences in edge weights between the networks of the two groups (*P* > 0.05).

## 4 Discussion

This is the first study to systematically compare the network structure of anxiety and depression between frail and non-frail elderly individuals. The NCT results showed no significant difference in the overall network structure of anxiety and depression between the frail elderly group and the control group. Although a slight decrease in overall network strength was observed in the non-frail elderly group, this difference was not statistically significant. On the contrary, both groups showed similar network characteristics regarding bridging symptoms, central symptoms, overall strength, and network structure.

Network analysis findings indicate that the central symptoms are identical between frail and non-frail elderly populations. However, the centrality of “ Feeling depressed or down “ (CESD3) was slightly higher in frail elderly individuals (EI = 1.20), compared to 1.17 in non-frail elderly individuals. Research has found that depression and frailty may share the same pathophysiological mechanisms. Elderly people with depression and frailty have elevated levels of inflammatory cytokines, which can directly affect muscle mass and muscle strength. They may also lead to the clinical manifestations of frailty, such as decreased muscle strength and function, by influencing the central nervous system and cardiovascular system. (40) Frailty and depression have bidirectional effects and can act as predictors of each other (41). The physical functions of frail elderly people decline and their activities decrease, which intensifies their depressive mood and leads to an increase in feelings of sadness.

GAD2 (Being unable to stop or control worrying) and GAD4 (Feeling tense and having difficulty relaxing) were both significant core symptoms in both groups, reflecting the widespread uncertainty and anxiety among the elderly in coping with stressors such as aging, illness, and widowhood. Sadness and worry may be common emotional responses to various life stresses and health problems. Cognitive behavioral therapy and relaxation therapy can help the elderly identify and change negative thinking patterns, develop skills for managing stress and emotions, and thereby better control symptoms of anxiety and depression (42).

In both groups, the common bridging symptoms were GAD1 (“Feeling anxious, worried, or distressed”), CESD1 (“Getting upset over small matters “). GAD7 (“Feeling as if something terrible might happen”) and GAD3 (“Worrying too much about various things “) were the bridging symptoms specific to the frail group and the non-frail group, respectively.

Modifying lifestyle habits, alleviating tension or anxiety, and reducing negative emotions stemming from being troubled can mitigate the comorbidity of anxiety and depression among the elderly. Within the two network models, GAD1 (“Feeling anxious, worried, or distressed”) demonstrated the closest association with CESD10 (“Having good sleep quality”) in the depression cohort. In the anxiety cohort, CESD1 (“Getting upset over small matters “) exhibited the strongest correlation with GAD6 (“Becoming easily annoyed or irritable “). The prevalence of multidimensional frailty is higher among elderly people with sleep disorders (43). Sleep disruption is a core feature of anxiety/anxiety-related disorders, and anxiety often worsens sleep quality, which speaks to a spurring of a negative cycle involving poor sleep and anxiety (44). Sleep disruption is a core feature of anxiety/anxiety-related disorders, and anxiety often deteriorates sleep quality, indicating that the negative cycle between poor sleep quality and anxiety is intensifying. Elderly people should be encouraged to exercise regularly, especially in combination with proper nutrition, which can help improve sleep, maintain muscle strength, mobility and quality of life (45).

Initiating with aerobic exercises, a moderate - intensity continuous training regimen can effectively stimulate muscle contractions during activities of daily living. This approach not only reduces the likelihood of fatigue and falls but also enhances the elderly’s motivation for physical activity. Moreover, it can improve frailty status and alleviate adverse emotions such as anxiety and depression (46, 47).

In the network of the “frail” group, among the depressive population, GAD7 (Feeling as if something terrible might happen) shows the closest association with CESD8 (Feeling lonely). Elderly people with low scores in interpersonal relationships may develop depressive symptoms due to loneliness and helplessness over a long period of time. Depressive symptoms, in turn, can affect their social skills, making it even more difficult for them to establish and maintain healthy interpersonal relationships, thus creating a vicious cycle. (48) The transition of social roles and the decline of physiological functions among the elderly inevitably result in social losses, exposing them to the risk of negative life events. If the elderly can promptly obtain external support from family members, friends, society, and the government, it can have a positive impact on the attitude and behavior of the elderly in the community when facing negative life events (49).

Both frail and non - frail elderly individuals are advised to embrace a healthy lifestyle, engage in regular physical exercise, participate in social activities, and consume fresh fruits and vegetables on a regular basis. Moreover, family members and community service providers should devote greater attention and care to the elderly to mitigate negative emotions.

This finding is inconsistent with previous studies, and several factors may contribute to this discrepancy. First, it is well known that physiological reactivity to stress diminishes with age while coping skills improve due to greater maturity and resilience (50, 51). Second, the mechanisms linking frailty to mental health may be more pronounced in middle age, such as hormonal changes or inflammation, which have a significant impact on mental health during this period (52). Later, these physiological systems become less active, resulting in a reduced influence on mental health (53). The lack of association in the elderly population may be attributed to the presence of multiple competing pathways for the development of frailty in later life. Additionally, chronic somatic diseases and anxiety disorders are prospectively correlated bidirectionally (54). Furthermore, the diagnostic accuracy of anxiety disorders may decrease with age, because it is challenging to distinguish symptoms caused by anxiety from those due to chronic somatic conditions (55).

The variability in definitions and methodological approaches among researchers may influence the results observed in the literature. In this study, both prefrailty (1 point) and robustness (0 points) are categorized as non-frailty states, which may affect the findings of this study. Data from a US prospective cohort indicate that when frailty is defined more broadly to encompass physical, nutritional, cognitive, and sensory dimensions, depression significantly increases the risk of frailty (56).

Several studies have shown that the association between frailty and depression is significant in various populations, including the general population, elderly clinic attendees, and those assessed using the Fried frailty phenotype, FRAIL scale, and GDS. However, this association is not significant in community-dwelling older adults or in studies utilizing the CES-D. Subgroup analyses based on study populations revealed no significant relationship between depression and frailty in community-dwelling elderly individuals. This discrepancy may be attributed to the substantial differences and high heterogeneity observed between the results of Figueiredo et al. and other studies. Additionally, changes in the depression scale can influence the prevalence of depression in frail elderly individuals; therefore, comparisons between subgroups are statistically critical (57).

This study has several strengths, including network analysis, PSM to ensure homogeneity of sample characteristics, and using a large, nationally representative database. However, there are also several limitations. This study employed a cross-sectional design, which precludes causal inferences due to the inability to establish the temporal relationship between frailty and mental health symptoms. Future research should prioritize longitudinal designs to systematically track symptom trajectories in older adults before and after spousal loss, elucidate the dynamic evolution of symptom networks over time, and ultimately inform the development of targeted long-term psychological interventions.Secondly, the sample comprises community-dwelling older adults aged ≥65 with a mean age over 92 years, limiting generalizability to younger elderly populations or non-Chinese cohorts. Future research could expand the age range of the sample and conduct cross-cultural comparisons to explore the differences in the impact of frailty on the mental health of the elderly at different age stages and in different cultural contexts.Thirdly, self-reported measures (CESD-10, GAD-7) may introduce recall bias, and frailty assessment using the modified SOF index (focusing on physical factors) may overlook cognitive or sensory dimensions. Furthermore, prefrailty (1 point) and robustness (0 points) are categorized as non-frailty states, which may affect the findings of this study. Fourthly, network analysis relies on cross-sectional data, failing to capture dynamic symptom changes over time. Lastly, the study does not account for comorbidities or environmental stressors, which may confound results.

Compared with the control group, elderly individuals with frailty exhibit more severe anxiety and depression. However, there are no significant differences in the network strength and structure of anxiety and depression between the two groups. Therefore, psychological and social interventions targeting central symptoms such as CESD3 (“Feeling depressed or down”; EI: 1.20), GAD2 (“Being unable to stop or control worrying”; EI: 1.13), and GAD4 (“Feeling tense and having difficulty relaxing”; EI: 1.07), as well as bridging symptoms such as GAD1 (“Feeling anxious, worried, or distressed”; BEI: 0.52), CESD1 (“Getting upset over small matters”; BEI: 0.42), GAD7 (“Feeling as if something terrible might happen”; BEI: 0.32), and GAD3 (“Worrying too much about various things”; BEI: 0.31) are equally beneficial for both frail and non-frail elderly populations.

In conclusion, the results of this study show that the presence or absence of frailty may not alter the relationship between these symptoms or their overall severity. However, psychosocial intervention measures aimed at improving anxiety and depression are equally effective for both frail and non-frail elderly individuals. Therefore, medical staff can develop more relevant intervention strategies to help allocate medical resources rationally and prevent or alleviate the occurrence of adverse mental health problems in the elderly.

## Data Availability

The original contributions presented in the study are included in the article/**Supplementary Material**. Further inquiries can be directed to the corresponding authors.
