# Supplementary material for: The relationship between frailty in older adults and anxiety and depression in china: propensity score matching and network analysis
Source: Front Psychiatry. 2025 Jul 30;16:1596015. doi: 10.3389/fpsyt.2025.1596015 (PMC12345367; doi:10.3389/fpsyt.2025.1596015)

**Supplementary materials**

Table S1. Specific values of the edge weights of networks in the frailty group and the non-frailty group

Table S2. Specific values of nodes EI and BEI of networks in the frailty group and the non-frailty group

Table S3. Specific values of the predictability of networks in the frailty group and the non-frailty group

Figure S1. Flowchart of sample selection

Figure S2. Distribution and histogram of propensity scores for the “frailty” group and the “non-frailty” group before and after matching

Figure S3. Accuracy of the edge weights

Figure S4. Network stability of nodes EI and BEI for the widowed group and the non-widowed group

Figure S5. Test of differences between node EI and the edge weights

Table S1. Specific values of the edge weights of networks in the frailty group and the non-frailty group

Table S2. Specific values of nodes EI and BEI of networks in the frailty group and the non-frailty group

Table S3. Specific values of the predictability of networks in the frailty group and the non-frailty group

Figure S1. Flowchart of sample selection

People participate CLHLS 2017-2018 wave (N=15,874)

Participants aged 65 years old and above (N=15,779)

People with complete assessment of frailty, anxiety, depression, and main demographic information (N=9,255)

PSM by age, gender, education level, marital status (widowed or not), living arrangements, daily sleep duration, medical payers, life satisfaction, smoking, drinking, taste preference, past and current exercise habits, with a ratio of 1:1 between the widowed group and the non-widowed group (N=1,754)

Non-widowed group (877)

Widowed group (N=877)

Figure S2. Distribution and histogram of propensity scores for the “frailty” group and the “non-frailty” group before and after matching


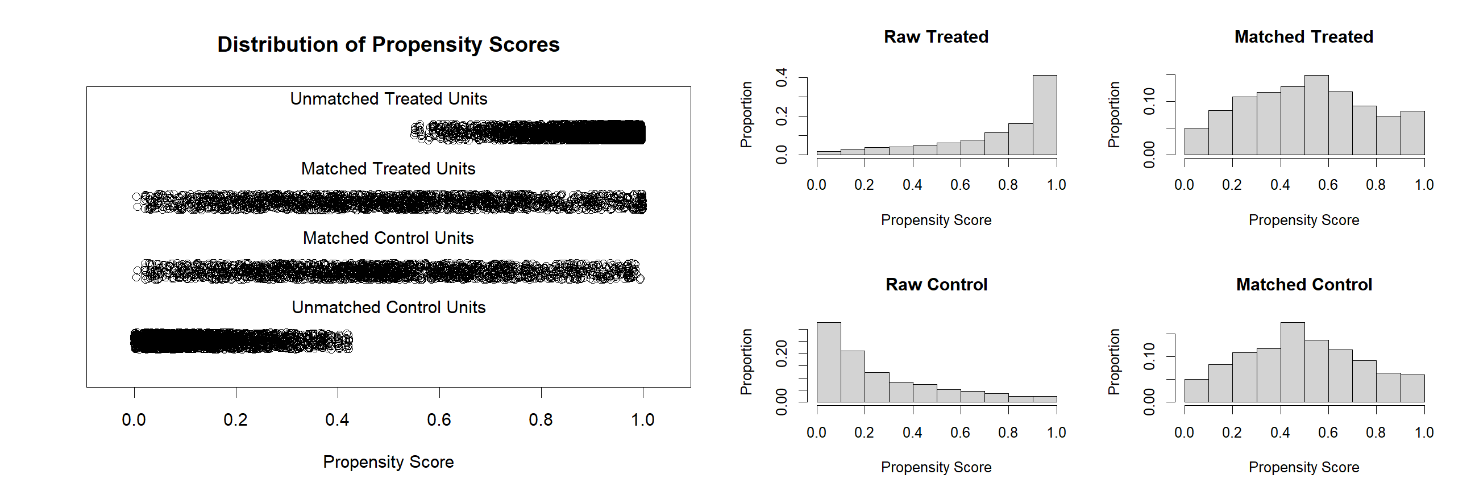


Figure S3.Accuracy of the edge weights
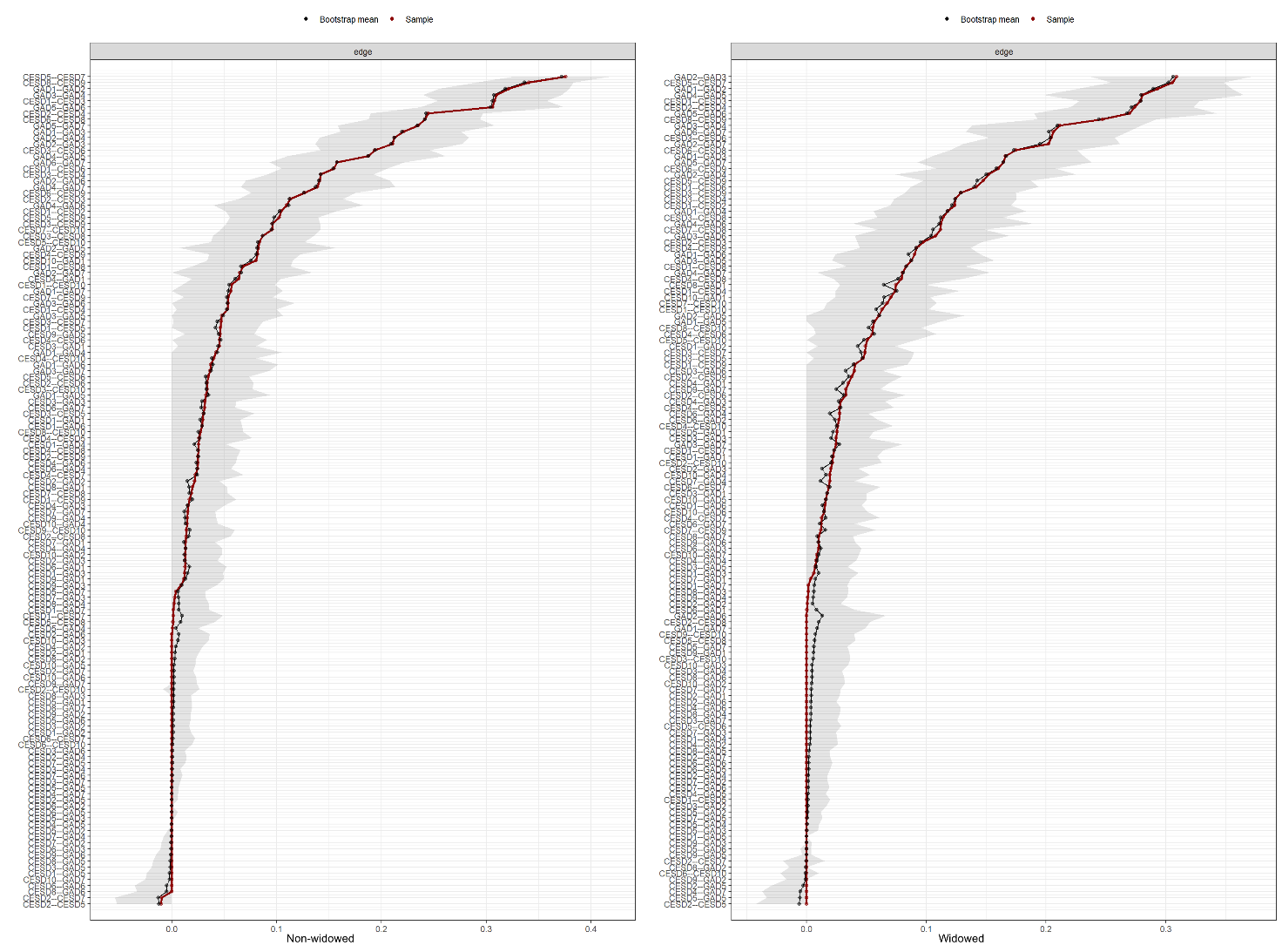


Figure S4. Network stability of nodes EI and BEI for the widowed group and the non-widowed group


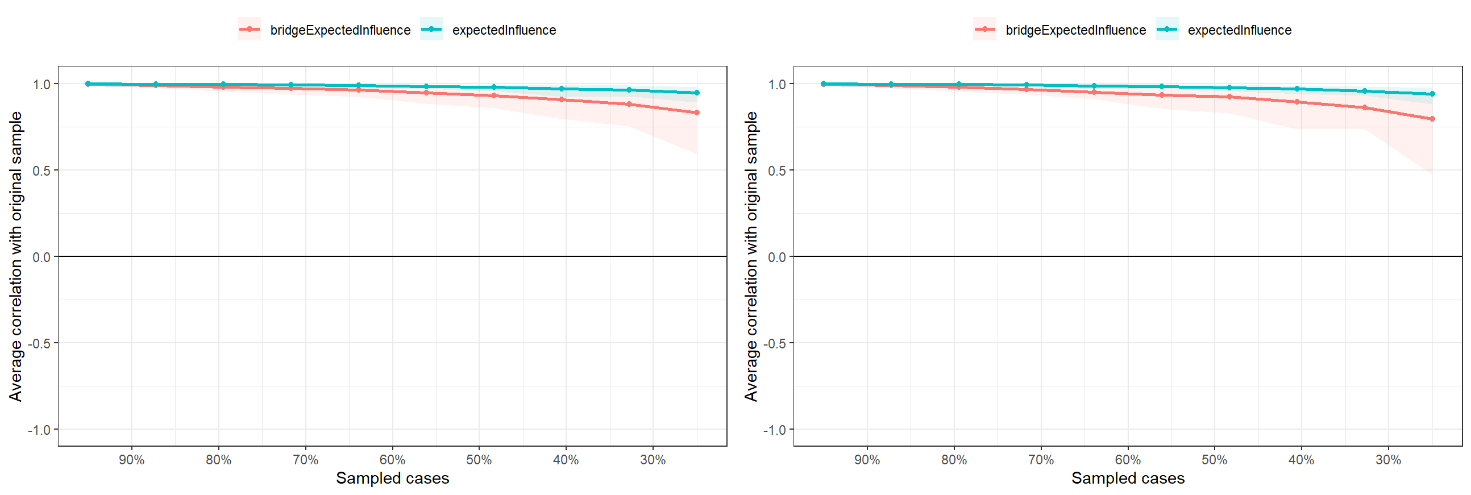


Figure S5. Test of differences between node EI and the edge weights


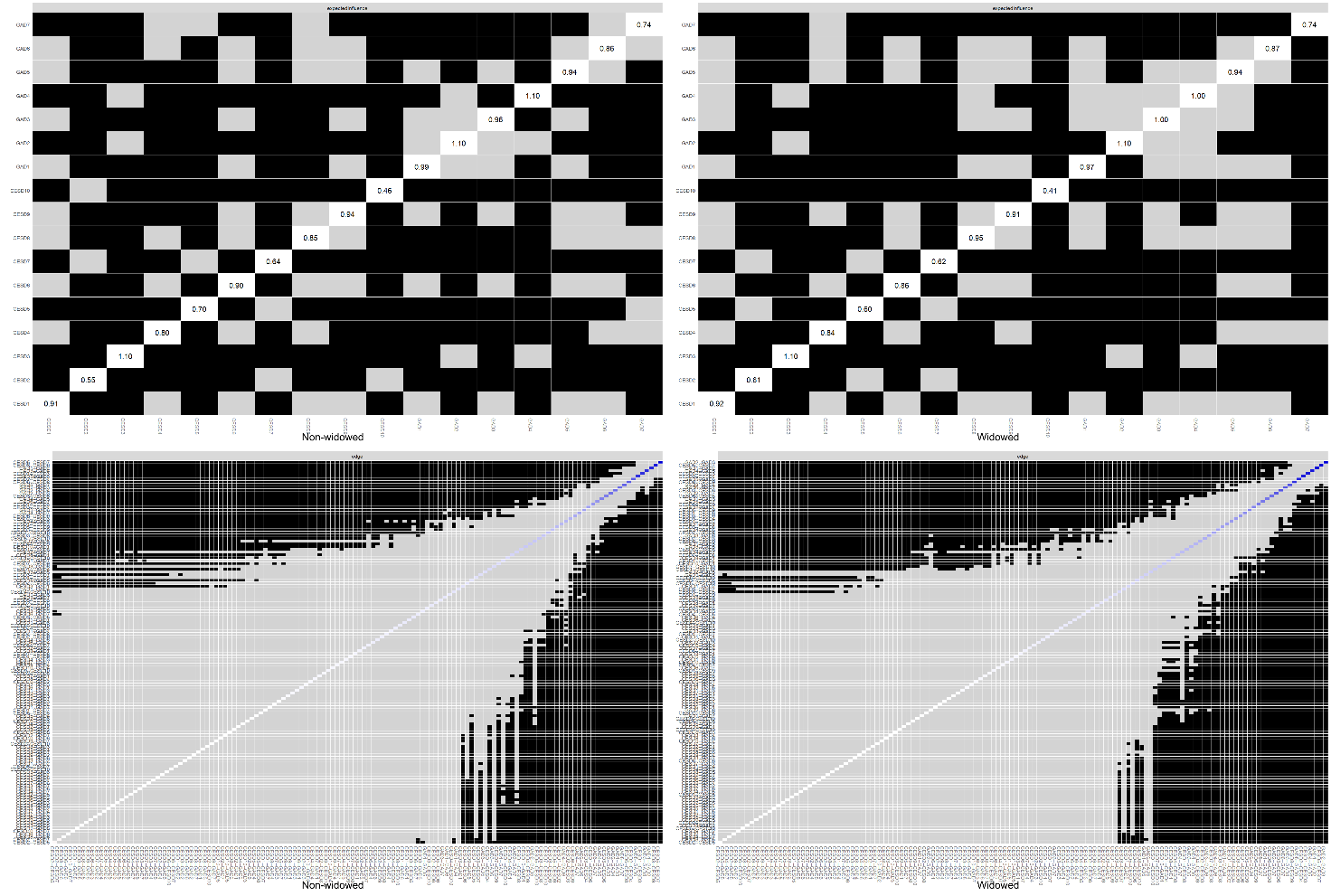

Supplement: Supplementary file 1 [file Supplementaryfile1.docx]
